# More than chronic pain: behavioural and psychosocial protective factors predict lower brain age in adults with/at risk of knee osteoarthritis over two years

**DOI:** 10.1093/braincomms/fcaf344

**Published:** 2025-09-11

**Authors:** Jared J Tanner, Angela Mickle, Udell Holmes, Brittany Addison, Kenia Rangel, Cynthia Garvan, Roland Staud, Song Lai, David Redden, Burel R Goodin, Catherine C Price, Roger B Fillingim, Kimberly T Sibille

**Affiliations:** Department of Clinical and Health Psychology, College of Public Health and Health Professions, University of Florida, Gainesville, FL 32610, USA; Department of Physical Medicine and Rehabilitation, University of Florida, Gainesville, FL 32610, USA; Pain Research & Intervention Center of Excellence, University of Florida, Gainesville, FL 32610, USA; Department of Clinical and Health Psychology, College of Public Health and Health Professions, University of Florida, Gainesville, FL 32610, USA; Department of Physical Medicine and Rehabilitation, University of Florida, Gainesville, FL 32610, USA; Department of Clinical and Health Psychology, College of Public Health and Health Professions, University of Florida, Gainesville, FL 32610, USA; Department of Anesthesiology, College of Medicine, University of Florida, Gainesville, FL 32610, USA; Department of Medicine, College of Medicine, University of Florida, Gainesville, FL 32610, USA; Department of Radiation Oncology & CTSI Human Imaging Core, University of Florida, Gainesville, FL 32610, USA; Department of Biostatistics, University of Alabama at Birmingham, Birmingham, AL 35233, USA; Department of Anesthesiology, Washington University, St. Louis, MO 63110, USA; Department of Clinical and Health Psychology, College of Public Health and Health Professions, University of Florida, Gainesville, FL 32610, USA; Department of Anesthesiology, College of Medicine, University of Florida, Gainesville, FL 32610, USA; Pain Research & Intervention Center of Excellence, University of Florida, Gainesville, FL 32610, USA; Department of Community Dentistry and Behavioral Science, University of Florida, Gainesville, FL 32610, USA; Department of Physical Medicine and Rehabilitation, University of Florida, Gainesville, FL 32610, USA; Pain Research & Intervention Center of Excellence, University of Florida, Gainesville, FL 32610, USA

**Keywords:** chronic musculoskeletal pain, behavioural/psychosocial protective factors, socioenvironmental risk, brain age, machine learning

## Abstract

The interplay between chronic musculoskeletal pain and brain ageing is complex. Studies employing machine learning models to assess relationships between brain age and chronic pain generally show that higher chronic pain severity associates with older brain age. Analyses to date have not considered individual and community-level socioenvironmental risk factors or behavioural/psychosocial protective factors as potential modifiers of cross-sectional and longitudinal brain age. This study aimed to elucidate the relationships between chronic pain, socioenvironmental risk, behavioural/psychosocial protective factors, and brain ageing. The sample comprised 197 adults (Men:Women = 68:129) from a prospective observational cohort study. Most individuals reported knee pain and were with/at risk of osteoarthritis. A subset of 128 participants (Men:Women = 41:87) completed a follow-up MRI session at 2 years and were included in the longitudinal analysis (Aim 2). Participants were 45–85 years of age and self-identified as non-Hispanic Black or non-Hispanic White. Data collected included demographics, health history, pain assessments, individual and community-level socioenvironmental factors (education, income, household size, marital and insurance status, and area deprivation index) coded as a summative socioenvironmental risk variable, and behavioural/psychosocial factors (tobacco use, waist circumference, optimism, positive and negative affect, perceived stress, perceived social support, sleep) coded as a summative behavioural/psychosocial protective factor variable. Structural MRI data were used to estimate brain age by applying a machine learning approach (DeepBrainNet). Cross-sectional analyses utilized regression and analysis of variance, while longitudinal analyses utilized a linear mixed model. Higher chronic pain stage and socioenvironmental risk are associated with an increased brain age gap (the difference between chronological age and predicted brain age). Participants who had higher socioenvironmental risk had brains that were about three years older than those of participants with lower risk. Having more behavioural/psychosocial protective factors correlated with a lower brain age gap; participants with higher behavioural/psychosocial protective factors had brains that were over three years younger than participants with fewer behavioural/psychosocial protective factors. Longitudinally, higher baseline behavioural/psychosocial protective factors are associated with lower brain age over the 2-year span, beyond the effects of chronic pain stage and socioenvironmental risk. Our findings show behavioural/psychosocial protective factors may counteract neurobiological ageing and help buffer the brain from chronic pain.

## Teaser

Tanner *et al*. report that higher chronic pain severity and socioenvironmental risk are linked to older brain age, while modifiable protective factors show a brain-sparing effect over a 2-year period. Fostering protective factors might mitigate neurobiological ageing in middle-age and older individuals with chronic musculoskeletal pain.

## Graphical Abstract

**Graphical Abstract:**
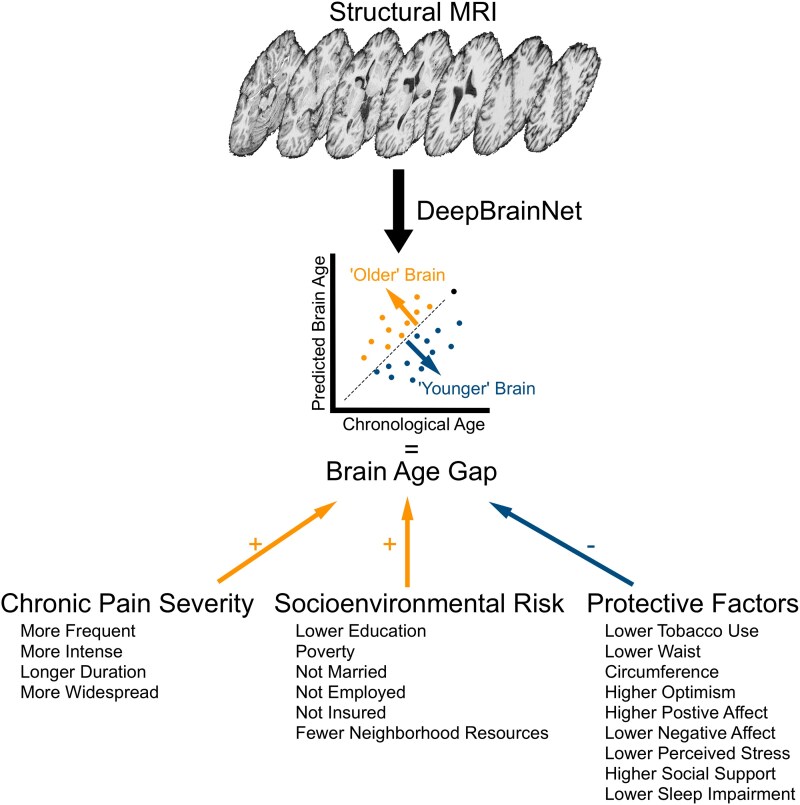

## Introduction

Chronic musculoskeletal (MSK) pain conditions are a leading cause of functional decline and disability worldwide and result in a significant societal burden.^1-3^ The biological consequences of chronic pain are also concerning, with evidence indicating cortical and subcortical brain differences in individuals with chronic MSK pain conditions compared to pain-free controls.^4-11^ Complicating matters, there is significant variability across clinical outcome measures in individuals with chronic MSK pain. Although brain imaging research has shown general associations with localized brain regions, chronic MSK pain outcomes are influenced by the experiences of the whole person.^12,13^ As such, we should expect not only localized but also widespread neurobiological associations with chronic MSK pain.

One approach to assess whole brain structure is through machine learning brain age models. Brain age is viewed as a proxy for both chronological and neurobiological age. Specifically, the difference between brain age and chronological age represents a comprehensive measure of ageing across the whole brain.^14,15^ To date, brain age models have been successfully applied to various health and neurological conditions^16-18^ including dementia,^19,20^ depression,^21,22^ and schizophrenia.^23^ They have also been applied to chronic pain, with mixed but mounting evidence for advanced global brain age associated with chronic pain severity.^24-29^ Brain age investigations, however, have been predominately cross-sectional, which limits conclusions about accelerated brain ageing.^30^ Longitudinal studies are needed to investigate machine learning brain age models to differentiate between brain ageing in healthy and clinical populations.

While there is a burden associated with chronic pain, the effects of pain, including functioning, can be buffered or exacerbated by socioenvironmental and behavioural/psychosocial risk or protective factors.^7,31-33^ Risk and protective factors have a biological interface. We and others have shown individual- and community-level socioenvironmental factors are associated with differences in chronic pain symptoms, a clinical multisystem composite, and brain structure.^34-41^ Individual-level socioenvironmental factors include education, income, number of people living in the household, and marital status. Adults with greater socioenvironmental risk (e.g. income at or below $25,000, less than a high school education, single without a spouse or partner) experience more disabling pain compared to those with lower socioenvironmental risk (SE risk).^34-36,38^ While many effects of SE risk are evident in early childhood development,^42,43^ the influence is also observed in middle-aged and older adults in both brain and functional outcomes.^37,44,45^

The Area Deprivation Index (ADI) is a community-level socioenvironmental factor reflecting resources across residential environments.^46,47^ The ADI has been associated with poor quality sleep, greater inflammatory measures, and worse chronic pain symptoms in individuals with chronic low back pain.^48,49^ The ADI also associates with brain structure^50^ and many chronic health conditions.^51,52^ The relationship is particularly pronounced for populations living in neighbourhoods in the highest ADI quintile, indicating greater deprivation.^53,54^

Individual behavioural/psychosocial factors can also promote pain-related risk or protection. We and others have investigated individual-level psychological and social factors (e.g. positive affect, negative affect, active coping, optimism, acceptance, understanding a purpose in life, and social support) and behavioural factors (e.g. physical activity, diet, maintaining a healthy weight, and being a non-smoker) in relation to chronic pain symptoms and biological measures.^7,31,55-64^ Behavioural/psychosocial protective factors are linked with better clinical, biological and other health outcomes in individuals with chronic MSK pain, including lower morbidity and mortality.^31,55-60,65^ Modifiable behavioural/psychosocial protective factors might buffer the neurobiological consequences of chronic pain and ageing.^7^

The current study aims to investigate the whole-person combination of socioenvironmental risk and behavioural/psychosocial protective factors and chronic pain severity on advanced global brain age. Specifically, while prior research suggests associations between chronic pain severity and advanced brain age,^18-21^ it is unclear how the combined contributions of socioenvironmental risk and behavioural/psychosocial protective factors in the context of chronic MSK pain are associated with global brain structure both cross-sectionally and longitudinally.

The purpose of this study is to determine, in a group of individuals with and without chronic MSK pain: (i) the relationships between chronic pain severity, socioenvironmental and behavioural/psychosocial factors,^7,61^ and brain age, and (ii) if behavioural/psychosocial protective factors predict brain age over a two-year period beyond the potential effects of chronic pain severity and socioenvironmental factors. We hypothesize (1a) higher chronic pain severity will associate with more advanced brain age, (1b) higher SE risk will associate with higher brain age, (1c) higher behavioural/psychosocial protective factors will associate with lower brain age, and (1d) in a combined model, higher behavioural/psychosocial protective factors will associate with lower brain age, reducing the ageing burden of greater chronic pain severity and higher SE risk. We also hypothesize that (2) behavioural/psychosocial protective factors will buffer against brain ageing over a two-year span.

## Materials and methods

### Ethics and consent

This study was performed in accordance with the Declaration of Helsinki. The UPLOAD-2 study received approval from the Institutional Review Board at the University of Florida (IRB approval number: 201400209) on June 6, 2014, and from the Institutional Review Board at the University of Alabama at Birmingham (IRB approval number: 40915002) on November 11, 2014. All participants provided both verbal and written informed consent before any study procedures were carried out.

### Study overview

The data for the study were from a prospective observational cohort study titled *Understanding Pain and Limitations in Osteoarthritic Disease-2* (*UPLOAD-2).* Collecting a comprehensive array of biopsychosocial factors, UPLOAD-2 was designed to better understand factors contributing to differences in health outcomes among adults with or at risk of knee osteoarthritis (OA). Data were collected between August 2015 and May 2017 at the University of Florida (UF) and the University of Alabama at Birmingham (UAB). The Institutional Review Boards at UF (IRB # 201400209) and UAB (IRB # 40915002) reviewed and approved all procedures. Written and verbal informed consent was obtained from all participants. Participants were recruited from clinic settings and through community advertisement, including radio, newspaper ads and posted flyers. The manuscript follows the STROBE Checklist Reporting Guidelines.^66^

### Participants

Baseline data were collected from 253 community-dwelling adults, 45–85 years old who self-identified as non-Hispanic White/Caucasian/European (NHW) or non-Hispanic Black/African American (NHB). Participant exclusion criteria included rheumatoid arthritis, fibromyalgia, knee replacement surgery, chronic daily opioid use, uncontrolled hypertension, cardiovascular or peripheral arterial disease, neurological diseases (e.g. multiple sclerosis), or psychiatric disorder necessitating hospitalization in the prior 12 months, and pregnancy or nursing. Of the 253 participants, 40 did not complete neuroimaging due to claustrophobia or safety concerns. We excluded 16 additional participants due to poor image quality, neuropathology, or missing covariates. The final sample for cross-sectional analyses included 197 participants (Men:Women = 68:129). The longitudinal analysis (Aim 2) included those individuals who completed a 2-year follow-up MRI session, totalling 128 participants (Men:Women = 41:87). Only procedures germane to the current investigation are described.

### Procedures

Following a standardized screening procedure, eligible participants attended a health assessment session (HAS) where they completed a number of questionnaires during and following the session specific to sociodemographics (e.g. education level), health behaviours (e.g. tobacco usage), pain and physical limitations, and psychosocial functioning. A waist measurement (in centimetres) was also obtained. Participants returned within approximately two weeks following the HAS to complete brain imaging. Visits were repeated two years later, following the initial baseline sessions (time point 2).

### Measures

#### Clinical pain and functioning

Chronic pain stage is a measure of chronic pain severity that has been shown to be sensitive to different biological measures, including clinical multisystem composite, telomere length (a measure of cellular ageing), and brain structure in temporal and frontal regions.^8,65,67^ Chronic pain stage is based on four domains of pain: frequency, intensity, duration, and total pain sites. *Frequency of pain* was determined based on the number of days per week that knee pain is experienced. *Intensity of pain* was determined by the Graded Chronic Pain Scale (GCPS) characteristic pain intensity. The GCPS assesses global knee pain severity over the past 6 months.^68^ Characteristic pain intensity is computed by averaging three measures: the intensity of current, average, and worst clinical pain over the past 6 months, from 0 (no pain) to 10 (pain as bad as it could be), and then multiplying by 10 (resulting in a range from 0 to 100). *Pain duration* is the length of time (months/years) the participant experienced knee pain. The *number of total pain sites* is a summation of the number of body sites (28 maximum, with 14 on each side) in which the participants indicate experiencing pain more days than not over the past 3 months.

A 0 or a 1 is assigned for each domain based on whether the value is below or above the median score; ranges are consistent with prior publications.^67^ The four domains are summed for the total score. Stages of chronic pain range from 1 to 5, with 1 indicating no to low chronic pain and 5 indicating high/severe chronic pain.

#### Socioenvironmental risk

The socioenvironmental risk (SE risk) index was developed as previously described using recognized and established values as previously reported.^39,69^ Briefly, data to calculate the SE risk variable included self-reported educational attainment (high school or less = risk, greater than high school = protective^70^), poverty level from the 2021 United States federal poverty guidelines according to self-reported income and household number (at or below poverty = risk, above poverty = protective^70,71^), marital status (widowed, divorced, separate, or never married = risk, married, living with someone = protective^72-74^), employment status (laid off/on leave, looking for work, disabled, student, or other = risk, working or retired = protective^75^), insurance status (not insured = risk, insured = protective^76^), and the individual’s neighbourhood disadvantage using the national Area Deprivation Index^47^ (upper 20% = risk, lower 80% = protective^54,75,77,78^). Eight participants had data missing for one of the measures; missing data were imputed by replacing them with data from another time point. Each variable was assigned a 0 for protective or 1 for risk based on recognized values and then summed with a score ranging from 0 to 6. For *post hoc* analyses, the median score determined low risk = 0–2 and high risk = 3–6.

#### Behavioural/psychosocial protective factors

We included a combination of recognized protective behavioural and psychosocial factors and validated measures consistent with our previous investigations: tobacco use, waist circumference, optimism, positive and negative affect, perceived stress, social support, and sleep impairment.^7,61,79^ Three-level ordinal scoring (0, 1, and 2) was used based on validated or reported ranges for each measure. A total score was based on a summation of all measures (0–16 score) with higher scores indicating higher behavioural/psychosocial protective factors. Median split low and high groups were also created for additional analyses.

##### Tobacco use

Participants were asked, ‘Have you smoked at least 100 cigarettes in your entire life?’.^80^ ‘No’ responses were coded as 2, ‘Yes-previously smoked but do not currently’ were coded as 1, ‘Yes-currently smoke’ was coded as 0.

##### Waist measurement

Waist circumference was measured in centimetres using a standard measuring tape. For women, ≤80 was coded as 2, 80–88 coded as 1, and>88 was coded as 0. For men, ≤94 was coded as 2, 94–102 was coded as 1, and ≥102 was coded as 0 based on the World Health Organization cut points.^81^

##### Life Orientation Test-Revised

The Life Orientation Test-Revised (LOT-R) assesses dispositional optimism and pessimism.^82^ Higher scores indicate greater optimism (0–24 score range). Scores ≤13 were coded as 0, 13–18 were coded as 1, and ≥18 were coded as 2.

##### Positive and Negative Affect Schedule

The Positive and Negative Affect Schedule (PANAS) consists of 10 positively valanced items and 10 negatively valanced items.^83^ Scores range from 10 to 50, with higher scores indicating greater positive or negative valence. Groups were based on a tertial split. Negative PANAS <12.2 was coded as 2, 12.2–24 was coded as 1, and >24 was coded as 0. Positive PANAS >28.6 was coded as 0, 28.6–41.4 was coded as 1, and >41.4 was coded as 2. For those missing three or less items for either the positive or negative words, we imputed the missing values by taking the number of items on the scale, dividing it by the number answered, and then multiplying by the sum of the answered items.

##### Perceived Stress Scale

The Perceived Stress Scale (PSS) measures perceived stress over the previous month by asking for a rating on thoughts and feelings.^84^ Higher scores indicate greater perceived stress (0–40 scores). Scores ≤13 were coded as 2, 14–26 were coded as 1, and 27–40 were coded as 0. Imputation was completed for those missing no more than two questions using the within-subject mean of answered questions, imputing the missing question(s).

##### The multidimensional scale of perceived social support

The Multidimensional Scale of Perceived Social Support (MSPSS) is a measure of perceived support from family, friends, and significant others.^85^ Scores range from 12 to 84, with higher scores indicating higher levels of perceived support. Scores 61–84 were coded as 2, 36–60 were coded as 1, and 12–35 were coded as 0. Imputation was completed for those missing no more than two questions using the within-subject mean of answered questions, imputing the missing question(s).

##### PROMIS sleep–related impairment—short Form 8a

The PROMIS sleep measures sleep disturbance and sleep-related impairment over the previous 7 days on a 1 (not at all) to 5 (very much) scale.^86^ Some questions were reverse-scored, so high scores correspond to greater sleep-related impairment. Scores were then summed for a total score range of 8–40. Scores ≤24 were coded as 2, 25–29 were coded as 1, scores >29 were coded as 0. Imputation was completed for those missing no more than two questions using the within-subject mean of answered questions, imputing the missing response(s).

### Brain imaging

Magnetic Resonance Imaging (MRI) was acquired using a 3 Tesla Philips Achieva (32-channel head coil at UF and 8-channel at UAB). For the present investigation, we used a high-resolution three-dimensional (3D) T1-weighted Magnetization-Prepared Rapid Gradient-Echo (MP-RAGE) sequence (TR/TE/α = 7.0 ms/3.2 ms/8^°^, 1 mm^3^ isotropic voxels, FOV: 240 × 240 × 176).

## MRI Processing

The MP-RAGE files were preprocessed using the Advanced Normalization Tools (ANTs) cortical pipeline script.^87^ We then registered the bias-corrected, skull-stripped files to the Montreal Neurological Institute (MNI) sixth -generation nonlinear 152 template^88^ using an affine registration with 12 degrees of freedom.^89-91^ For longitudinal analyses (Aim 2), MP-RAGE files were preprocessed using the *antsLongitudinalCorticalThickness* script. For the current analysis, we performed visual quality assessment at each step of processing to verify the accuracy of inputs and outputs. We also performed quality assessment of MRI using the Computational Anatomy Toolbox for SPM (CAT12)^92^ using the cross-sectional pipeline (Aim 1) and longitudinal pipeline (Aim 2). CAT12 provides a quantitative metric with values ranging from 0 to 1 summarizing the quality of MP-RAGE images. Higher values indicate better image quality.^92^

 

#### Predicted brain age

**Figure 1:**
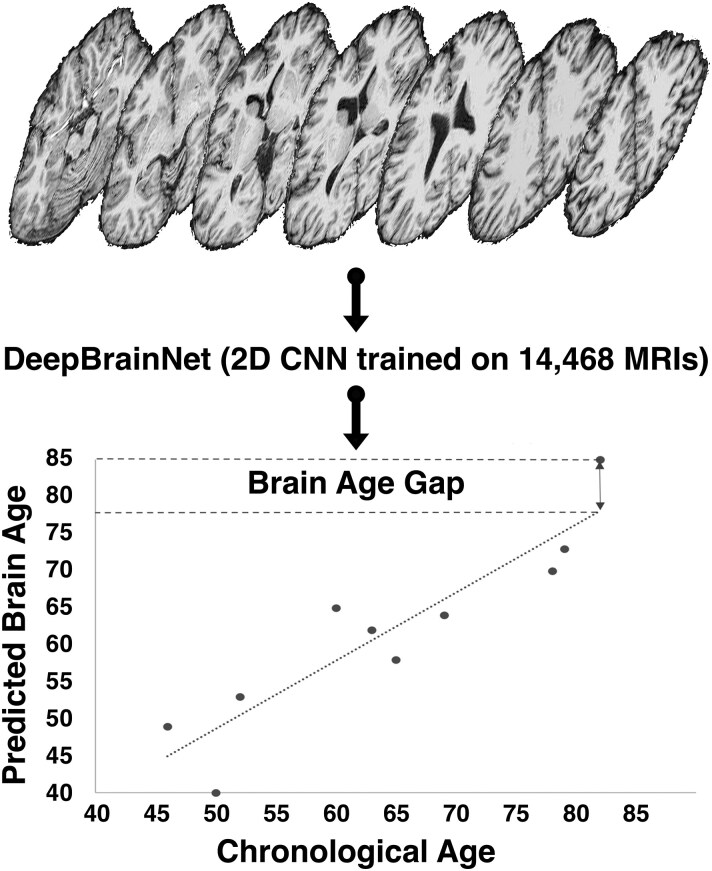
**An overview of the MP-RAGE processing with DeepBrainNet and calculation of brain age gap.** Caption: A summary of DeepBrainNet calculation of predicted brain age and brain age gap (BAG) from Magnetization Prepared Rapid Gradient Echo (MP-RAGE) Magnetic Resonance Imaging (MRI). The shown MP-RAGE axial slices are from one of our participants. Pre-processed MP-RAGE images for each participant were used for input into the pre-trained DeepBrainNet 2-Dimensional Convolutional Neural Network (2D CNN) model to yield a predicted brain age. The bottom scatter plot shows a representative relationship between predicted brain age and chronological age. The diagonal dotted line is a hypothetical perfect correlation between predicted brain age and chronological age. The difference between predicted brain age and chronological age (predicted brain age—chronological age) is the brain age gap. Positive values indicate an ‘older’ brain and negative values a ‘younger’ brain. This process was completed for all participants (*n* = 197).

Age predictions based on MRI are useful for understanding brain and body health.^16-18^ Machine learning brain age models have also been successfully applied to chronic pain.^24,26,28,93^ To address study questions related to chronic pain and behavioural/psychosocial protective factors, we calculated predicted brain age using an externally validated machine learning DeepBrainNet model that was based on T1-weighted MRIs from 14 468 individuals ages 3–95^17^ (Fig. 1). Briefly, after preprocessing as described previously, the MP-RAGE images were run through a publicly available Python-based script using the pre-trained DeepBrainNet model to generate predicted brain age for each participant.

#### Brain age gap

The difference between predicted brain age and chronological brain age correlates with various cognitive, behavioural, and health factors, including chronic pain.^93-98^ The brain—chronological age difference also differentiates between healthy adults and various clinical populations.^16,17,99^ To calculate the brain—chronological age difference, we subtracted chronological age from brain age to generate a brain age—chronological age gap (BAG; Fig. 1).^98,100^

### Variables of interest

Our main variables of interest were chronic pain stage, SE risk, behavioural/psychosocial protective factors, and brain age gap (BAG) for cross-sectional analyses (Aim 1) and predicted brain age for longitudinal analyses (Aim 2).

### Predictors of non-interest

Age is associated with both pain^101^ and brain changes.^102^ Age was thus included as a predictor of non-interest in analyses (either directly in the BAG variable or in the longitudinal model). As this included data from multiple sites, the study site (1 = UF, 2 = UAB) was also included as a predictor of non-interest. Total current comorbidities were selected from a pre-specified list including high blood pressure, heart disease, cancer, diabetes, asthma/breathing problems, kidney disease, thyroid problems, stroke, seizure, chronic pain, neurological disorder, depression, other mental health condition or other health problem (range: 0–14). Previous research demonstrates women, relative to men, have higher prevalence and severity of chronic MSK pain conditions.^103,104^ There are also sex group differences in the relationship between SE risk and behavioural/psychosocial protective factors and pain^7,105,106^ and sex differences in brain structure and neurodegenerative processes associated with advancing age.^7,16,107,108^ Thus, self-reported sex (1 = male, 2 = female) was included in statistical models. The CAT12 image quality rating (IQR) was previously shown to relate to brain structure metrics;^109^ therefore, we included it as a predictor of non-interest in analyses.

### Data analyses

We conducted data analyses in R version 4.3.2. Data were checked for normal distributions, outliers, and missing data. We previously described imputation for missing data. We also visually inspected the distributional form of the data. Testing of the distributions suggested the variables were not normally distributed (Shapiro-Wilk *P* values < 0.002). Outliers (>1.5 × the interquartile range) were identified in the following variables: comorbidities (high: *n* = 1), IQR (low: *n* = 5), and BAG (low: *n* = 3). Based on our sample size and the fact that there were no other patterns that would otherwise indicate a violation of assumptions, parametric statistics were used, and we did not exclude any participants. All tests were two-sided with statistical significance set at *P* < 0.05. We did not correct for multiple comparisons because of our limited and theory-grounded, targeted hypotheses.^110^

#### Statistical models

To address Aim 1, we performed three parallel multiple regression analyses to assess minimally adjusted relationships between (1a) chronic pain stage and BAG, (1b) SE risk and BAG, (1c) behavioural/psychosocial protective factors and BAG and (1d) behavioural/psychosocial protective factors and BAG accounting for chronic pain stage and SE risk. Our three primary predictors of interest (chronic pain stage, SE risk, and behavioural/psychosocial protective factors) are ordinal variables, but because each has at least five levels, they are approximations of continuous variables and thus treated as such in analyses.^111^ Effect sizes of primary predictors of interest were calculated using Cohen’s *f*2. Additionally, to investigate *post hoc* BAG differences with higher and lower levels of behavioural/psychosocial protective factors, median split high and low behavioural/psychosocial protective factors groups^112,113^ (median = 11; Lower behavioural/psychosocial protective factors < 11, Higher behavioural/psychosocial protective factors ≥ 11) and median split SE risk groups^39^ (low risk = 0–2, high risk = 3–6) were compared using ANCOVA. Effect sizes for the *post hoc* analyses are reported as $\eta_{P}^{2}$ with 90% confidence intervals.

To address Aim 2, we fit a linear mixed model by restricted maximum likelihood with t-tests using Satterthwaite’s method using the R package *lme4*. We used the following formula: predicted brain age ∼ time + sex + age + site + comorbidities + image quality rating + chronic pain stage + SE risk + behavioural/psychosocial protective factors + (1 | participant ID) + time* behavioural/psychosocial protective factors. The following variables were modelled as varying with time: (i) brain age, (ii) time as a categorical variable denoting time point, and (iii) image quality rating. The other variables (sex, site, comorbidities, chronic pain stage, SE risk, and behavioural/psychosocial protective factors) were modeled as time invariant. Age also kept as a time invariant to minimize standard errors due to overlap with the time variable. All predictive variables were modeled as fixed effects, except participant ID, which was allowed to vary randomly to capture individual differences that are not explained by the fixed effects. While our primary aim was to investigate the role of behavioural/psychosocial protective factor in brain age over two years, a *post hoc* analysis was run, consistent with Aim 1, to better understand the individual effects of baseline chronic pain stage, socioenvironmental risk, and behavioural/psychosocial protective factors on brain age over time.

## Results

### Descriptive results

Table 1 shows summaries of descriptive variables for the participants in our sample. The number of total pain sites, which is one component of the chronic pain stage, indicated a median of 4 total pain sites in the sample, with the knees (135/197), lower back (90/197), and hands (71/197) being the most frequently reported chronic pain sites. BAG correlated with chronological age (*r* = −0.16, *P*  *=* 0.025), sex (*r* = −0.28, *P* < 0.001), and IQR (*r* = −0.17, *P*  *=* 0.017).

**Table 1:** Summary statistics

Variable	Mean/Count	S.D.	Median	Min	Max
Age (years)	58.18	8.35	57.00	44.00	80.00
Sex (M:F)	68:129				
Study Site (UF:UAB)	124:73				
Comorbidities	1.14	1.13	1.00	0.00	7.00
Image quality rating	78.74	3.75	79.26	61.36	84.85
Chronic pain stage	2.87	1.49	3.00	1.00	5.00
Behavioural/psychosocial factors	10.60	2.86	11.00	3.00	16.00
SE Risk	1.98	1.73	2.00	0.00	6.00
Brain age gap (years)	−2.08	6.75	−1.92	−29.23	14.78
Time point 1 age (years)^a^	58.86	8.53	57.34	45.12	80.52
Time point 2 age (years)^a^	60.85	8.53	59.26	47.21	82.43
Time point 1 brain age (years)^a^	55.41	10.47	56.76	24.24	74.14
Time point 2 brain age (years)^a^	56.78	10.26	57.25	26.41	75.31

*N*  *=* 197. M = Male, F = Female. UF = University of Florida, UAB = University of Alabama at Birmingham, and SE Risk = socioenvironmental risk.

^a^These values are from the Aim 2 (longitudinal) sample: *N* = 128.

For the longitudinal aim (*n* = 128), predicted brain ages increased on average 1.37 years over the 2-year span of the study, with 31% of the sample having lower predicted brain ages at time point 2 compared to time point 1. A *post hoc* analysis comparing those who had brain age increase versus brain age decrease provided insufficient evidence of the unadjusted baseline predictors differing between participants who had increased or decreased predicted brain ages at time point 2 (*P* values > 0.05; see Supplementary Results).

#### Assessing the relationship between chronic pain stage and BAG

**Figure 2:**
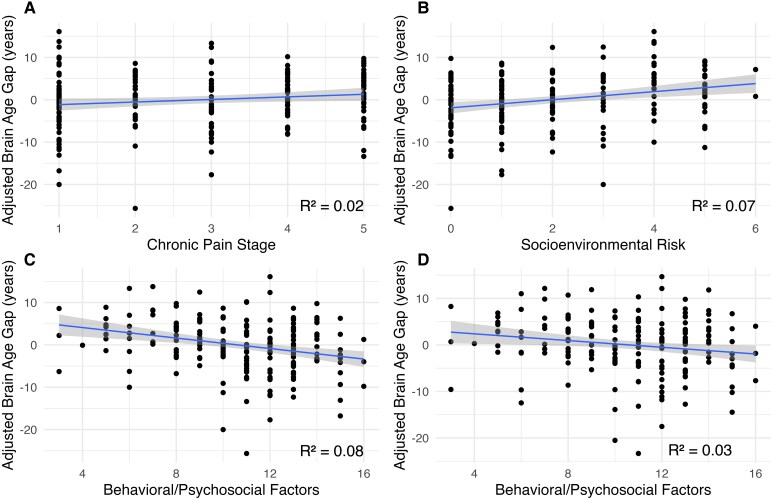
**Scatter plots depicting relationships between predictors of interest and brain age gap.** Caption: All relationships shown are statistically significant (Pearson *r* correlations, *P* values < 0.05; *n* = 197). Individual dots indicate the adjusted BAG values for each participant. (**A**) (Hypothesis 1a) depicts the relationship between chronic pain stage and brain age gap (BAG). BAG is adjusted for sex, study site, health comorbidities, and image quality rating. (**B**) (Hypothesis 1b) depicts the relationship between socioenvironmental risk and BAG, with BAG adjusted for the same variables as in **A**. Panel **C** (Hypothesis 1c) depicts the relationship between behavioural/psychosocial protective factors and BAG, with BAG adjusted for the same variables as in **A**. Panel **D** (Hypothesis 1d) depicts the relationship between behavioural/psychosocial protective factors and BAG, with BAG adjusted for the same variables as in **A** plus chronic pain stage and socioenvironmental risk.

In a regression model (*F*[5191] = 5.65, *P* < 0.001) adjusting for IQR, sex, study site, and comorbidities, higher chronic pain stage was associated with higher BAG (*P*  *=* 0.037; Cohen’s *f^2^* = 0.02; Fig. 2A; Supplementary Table 1). Specifically, each one-level increase in chronic pain stage corresponded to a 0.67-year increase in BAG.

#### Assessing the relationship between SE risk and BAG

In a regression model (*F*[5191] = 7.91, *P* < 0.001) adjusting for IQR, sex, study site, and comorbidities, higher SE risk (*P* < 0.001; Cohen’s *f*2 = 0.08) was significantly associated with higher BAG (Fig. 2B, Supplementary Table 2). Specifically, each one-level increase in SE risk corresponded to a 1-year increase in BAG. *Post hoc* group analyses showed those in the median split high SE risk group had adjusted BAG values that were 2.99 years higher than the low SE risk group (*t* = 3.273, *P*  *=* 0.001; $\eta_{P}^{2}=0.05$ [0.01, 0.11]; see Supplementary Fig. 1).

#### Assessing the relationship between behavioural/psychosocial protective factors and BAG

In a regression model (*F*[5191] = 8.69, *P*  *<* 0.001) adjusting for IQR, sex, study site, and comorbidities, higher behavioural/psychosocial protective factors (*P*  *<* 0.001, Cohen’s *f*2 = 0.09) were significantly associated with lower BAG (Supplementary Table 3, Fig. 2C). Specifically, each one-level increase in behavioural/psychosocial protective factors corresponded to a 0.69-year decrease in BAG. *Post hoc* group analyses showed those in the median split high behavioural/psychosocial protective factors group had BAG values that were 3.78 years lower than the low behavioural/psychosocial protective factors group (*t* = 4.10, *P*  *<* 0.001, $\eta_{P}^{2}$ = 0.08 [0.03, 0.15]; see Supplementary Fig. 2).

#### Assessing the relationship of a combined model of chronic pain stage, SE risk, behavioural/psychosocial protective factors and BAG

**Figure 3:**
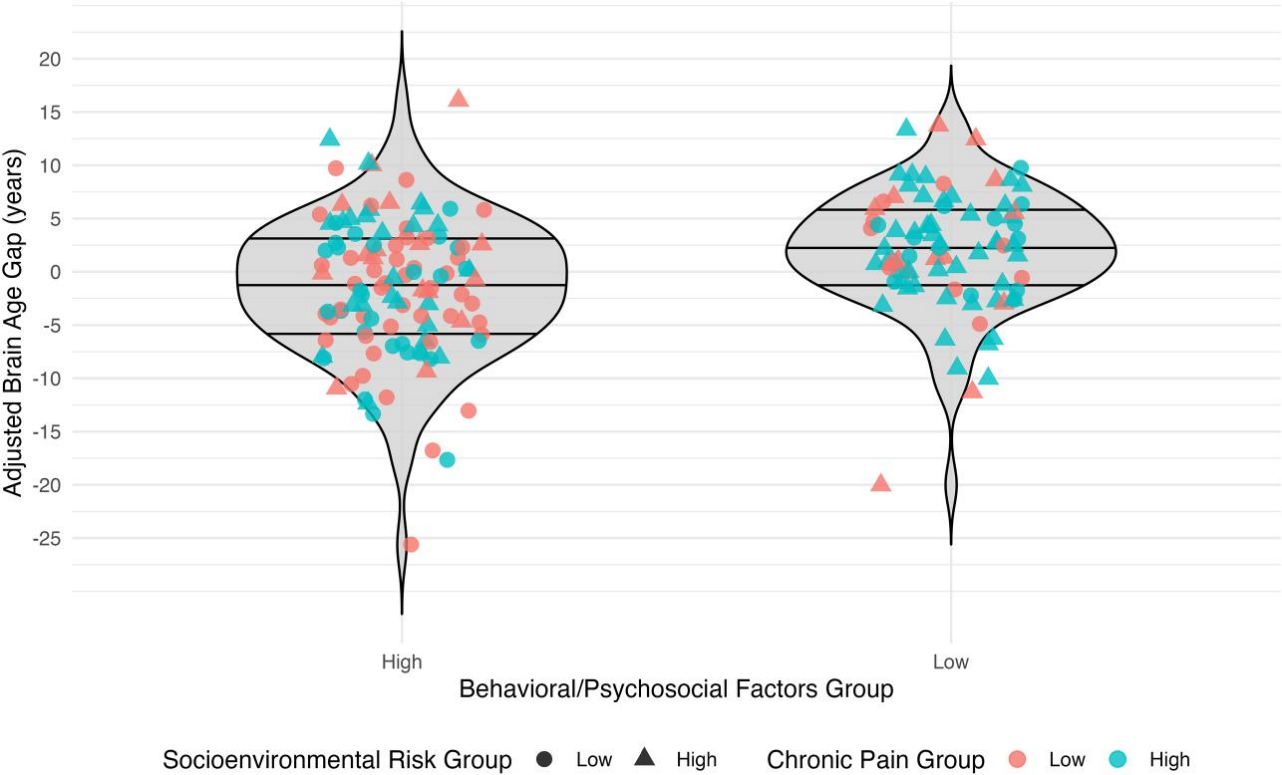
***Post hoc* behavioural/psychosocial protective factors median split group analysis violin plots.** Caption: All groups are median split. Shapes denote socioenvironmental risk group membership, and colors denote chronic pain group membership. Individual circles and triangles are the adjusted BAG values for each participant. When adjusting for sex, study site, health comorbidities, image quality, chronic pain stage, and socioenvironmental risk, the group with higher behavioural/psychosocial protective factors had an adjusted brain age gap (BAG) 2.69 years ‘younger’ than the group with low behavioural/psychosocial protective factors (Model ANCOVA F(7189) = 7.18, *P* < 0.001; group *t* = −2.70, *P*  *=* 0.008; *η_p_*^2^ = 0.04 [0.01, 0.09], *n* = 197).

In the combined regression model with chronic pain stage, SE risk, and behavioural/psychosocial protective factors, the overall model was significant (*F*[7189] = 7.25, *P*  *<* 0.001). Higher behavioural/psychosocial protective factors (*P*  *=* 0.006) and lower SE risk (*P*  *=* 0.029) were significantly associated with lower BAG (Table 2, Fig. 2D). Chronic pain stage was not significantly associated with BAG (*P*  *=* 0.369). The only predictor of non-interest that showed a significant relationship with BAG in this model was sex (females < males; *P*  *<* 0.001). *Post hoc* group analyses for the fully adjusted model (F(7189) = 7.18, *P* < 0.001) showed that those in the median split high behavioural/psychosocial protective factors group had BAG values that were 2.69 years lower than the low behavioural/psychosocial protective factors group (*t* = 2.70, *P*  *=* 0.008; $\eta_{P}^{2}$ = 0.04 [0.01, 0.09]). Figure 3 shows the *post hoc* depiction of median split behavioural/psychosocial protective factors groups considering chronic pain stage and SE risk.

**Table 2:** Full regression model predicting brain age gap

Predictor	*b*	95% CI	*beta*	95% CI	Unique *R*^2^	95% CI	*r*	Fit
(Intercept)	22.92	[−0.53, 46.37]						
IQR	−0.19	[−0.46, 0.09]	−0.10	[−0.26, 0.05]	0.01	[−0.01, 0.03]	−0.19**	
Sex	−3.26**	[−5.13, −1.39]	−0.23	[−0.36, −0.10]	0.05**	[−0.00, 0.10]	−0.28**	
Site	−1.09	[−3.14, 0.96]	−0.08	[−0.23, 0.07]	0.00	[−0.01, 0.02]	−0.01	
Comorbidities	−0.19	[−1.03, 0.65]	−0.03	[−0.17, 0.11]	0.00	[−0.01, 0.01]	0.05	
Chronic pain stage	0.29	[−0.34, 0.92]	0.06	[−0.08, 0.20]	0.00	[−0.01, 0.02]	0.17*	
SE Risk	0.63*	[0.07, 1.19]	0.16	[0.02, 0.31]	0.02*	[−0.01, 0.06]	0.31**	
Behavioural/Psychosocial Protective factors	−0.49**	[−0.85, −0.14]	−0.21	[−0.36, −0.06]	0.03**	[−0.01, 0.08]	−0.32**	
								*R* ^2^ = 0.212**
								95% CI[0.09,0.28]

*N* = 197. *b* = unstandardized regression weight. *beta* = standardized regression weight. Unique *R*^2^ = semipartial correlation squared. *r* = zero-order correlation.

CI = confidence interval. IQR = Image Quality Rating; SE Risk = socioenvironmental risk.

^*^ *P*  *<* 0.05; ** *P*  *<* 0.01.

#### Association between chronic pain stage, socioenvironmental risk, and behavioural/psychosocial protective factors and brain age changes over a 2-year period

We ran a linear mixed model (Table 3) to investigate brain ageing, adjusting for observation point (time), sex, age, study site, comorbidities, and IQR over a 2-year period. Of the primary predictors of interest, higher behavioural/psychosocial protective factors predicted lower average brain age over time (coefficient = −0.71; *P*  *<* 0.001). The interaction between time and behavioural/psychosocial protective factors was not significant (coefficient = −0.25; *P*  *=* 0.061), but the direction was suggestive of higher behavioural/psychosocial protective factors associating with slower brain ageing (see Supplementary Fig. 3). There was insufficient evidence that chronic pain stage (*P*  *=* 0.365) or SE risk (*P*  *=* 0.199) were associated with higher brain age over time in this model. For the predictors of non-interest, advancing time associated with higher brain age (*P*  *<* 0.001), higher chronological age associated with a higher brain age (*P*  *<* 0.001), female sex associated with nearly 3-year lower brain age (*P*  *=* 0.016) and higher image quality associated with a lower brain age (*P*  *=* 0.007).

**Table 3:** Linear mixed model predicting brain age over 2 years

Parameter	*Coefficient*	SE	95% CI	*t*	*P*
Fixed effects					
(Intercept)	36.41	10.97	[14.91, 57.91]	3.32	0.001
Time*	4.17	1.50	[1.24, 7.10]	2.79	0.006
Sex*	−2.96	1.22	[−5.35, −0.57]	−2.42	0.017
Age**	0.94	0.07	[0.81, 1.08]	13.70	0.000
Site	−0.77	1.19	[−3.11, 1.56]	−0.65	0.518
Comorbidities	0.18	0.57	[−0.94, 1.30]	0.32	0.752
IQR**	−0.30	0.11	[−0.52, −0.09]	−2.73	0.007
Chronic pain stage	0.36	0.39	[−0.41, 1.13]	0.91	0.366
SE Risk	0.46	0.36	[−0.24, 1.15]	1.29	0.200
Behavioural/Psychosocial protective factors**	−0.71	0.23	[−1.17, −0.25]	−3.03	0.003
Time*Behavioural/Psychosocial factors	−0.25	0.13	[−0.52, 0.01]	−1.89	0.062
Random effects					
Participant (intercept)	0.56	0.04	[0.48, 0.65]		
Residual (observations)	0.28	0.02	[0.25, 0.31]		

*N* = 128; SE = standard error of the model estimates; IQR = Image Quality Rating; SE Risk = socioenvironmental risk; Participant (Intercept) = the standard deviation of the random intercepts across participants; Residual (observations) = Residual standard deviation.

^*^ *P*  *<* 0.05; ** *P*  *<* 0.01.

In the *post hoc* linear mixed effects model analyses (see Supplementary Tables 4–6), we found the following: (i) without SE risk or behavioural/psychosocial protective factors in the model, higher chronic pain stage associated with higher brain age over the two-year period (*P* = 0.036), (ii) higher SE risk, without the other two predictors of interest in the model, associated with higher brain age over the two-year period (*P* = 0.022), and (iii) behavioural/psychosocial protective factors, without the other two predictors of interest, associated with higher brain age over the two-year period (*P* < 0.001).

## Discussion

In a sample of 197 middle-aged and older adults, the majority with chronic musculoskeletal pain, we found that those who reported higher chronic pain severity (Hypothesis 1a) had older brains; each one-level increase in chronic pain severity was associated with a 0.67-year increase in brain age gap (the difference between predicted brain age and chronological age). Additionally, adults who reported more socioenvironmental (SE) risk had a brain age gap that increased by one year for each level of SE risk (Hypothesis 1b).

Specific to Hypothesis 1c, significant associations were observed between behavioural/psychosocial protective factors and brain age gap. At baseline, adults who had higher behavioural/psychosocial protective factors had lower brain age gaps—0.69 years younger for each level of the 16-point scale. This was still significant when accounting for chronic pain stage and SE risk (Hypothesis 1d). Specifically, in the full linear regression model, participants reporting more widespread and severe chronic pain relative to those reporting the lowest, had brains that were about 1.5 years *older* (not statistically significant); participants with the highest SE risk (e.g. lower education, household income at/below poverty level) relative to the lowest had brains that were about 4.5 years *older*; and participants with the highest behavioural/psychosocial protective factors (e.g. more optimism, lower negative affect, less sleep impairment, lower waist circumference) relative to the lowest had brains that were about 8 years *younger* at baseline.

Regarding Hypothesis 2, higher behavioural/psychosocial protective factors were significantly associated with lower brain age over the 2-year timeframe. Additionally, while not statistically significant, each 1-unit increase in the behavioural/psychosocial protective factor slowed predicted brain ageing by about 0.25 years over 2 years. With behavioural/psychosocial protective factors in the model, neither chronic pain stage nor SE risk remained as significant predictors of brain ageing over the two-year window. Findings provide evidence that behavioural/psychosocial protective factors appear to serve as a buffer to the brain ageing process.

### Pain and brain age gap

Earlier literature has shown that higher chronic pain severity associates with less gray matter and other morphometric features.^4,6,10,11,114-116^ Typically, these studies focus on localized relationships, but reviews of literature suggest pain—brain relationships can span much of the brain.^117^ Regions of the brain directly implicated in chronic pain, however, are involved not only in pain processing but also in many diverse cognitive and affective processes. Our evidence of an association between a whole-brain summary metric (brain age) and clinical ratings of pain agrees with previous investigations of the relationships between brain age and chronic pain.^24-27,29,93^ One study using the same model of brain age (DeepBrainNet) found adults with osteoarthritis (including participants in the current study) had advanced brain ages relative to participants with chronic back pain and control participants without chronic pain.^93^ Our findings add to the existing literature by demonstrating that while chronic pain severity is associated with advanced brain age at baseline and over a 2-year period (Supplementary Table 4), the relationship between chronic pain and brain age was no longer statistically significant after considering socioenvironmental risk and behavioural/psychosocial protective factors (Table 2).

The modest effect we found between chronic pain stage and brain age is likely partially due to our sample characteristics. Our study included a sample of sociodemographically diverse participants, the majority of who had chronic musculoskeletal pain, most with mild to moderate knee pain, with less than 50% meeting knee OA criteria. Our previous research shows that more than chronic pain severity contributes to the biological heterogeneity observed in individuals with chronic musculoskeletal pain.^9,39,40^

Outside of chronic pain, there is a significant amount of literature linking brain age gap to mental and physical health, including cardiovascular risk factors,^118^ lifestyle factors,^15,119^ and typical ageing.^120^ While we did not account for all the factors, the brain age gap appears to be an excellent proxy that reflects the complex combination of life and health factors simultaneously.^29^

### Socioenvironmental risk and brain age gap

We have previously reported on relationships between socioenvironmental risk, chronic pain stage, and brain structure.^39-41^ Our findings add to the literature by demonstrating cross-sectional and longitudinal associations (Supplementary Table 5) between socioenvironmental risk and brain age gap; namely, those with higher risk had older appearing brains at baseline. However, with behavioural/psychosocial factors in the model, socioenvironmental risk did not predict brain age over the two-year span of our study.

It is reported that socioenvironmental risk influences on brain age can start early in life.^121^ One component of our socioenvironmental risk index is poverty. While the mechanisms of the relationships between socioenvironmental risk and brain age are not clear, poverty affects the developing brain^122^ and the risk of neurodegenerative disease in late life.^123^ Other research has linked advanced brain age with income and education.^124^ Income can provide experiences that serve as a stimulus and access to resources that are health-protective across the lifespan.^125^ There is also evidence of associations between family income in childhood and income in adulthood,^126^ family income and childhood brain development,^127^ and family income and health outcomes in adulthood.^128^

Our measure of socioenvironmental risk also included whether participants lived in neighbourhoods with the fewest resources, based on the Area Deprivation Index (ADI).^47^ Previous research shows living in neighbourhoods in the highest ADI quintile is associated with worse health outcomes.^53,54^ Neighbourhood disadvantage might serve as a proxy for various environmental and lifestyle factors that can influence cognition and dementia in late life (see for a review^52^). Prior to the inclusion of behavioural/psychosocial factors, socioenvironmental effects were significant for higher brain age over time (Supplementary Table 5). We speculate that socioenvironmental risk in combination with chronic pain and low behavioural/psychosocial protective factors would contribute to increased risk of future dementia.^9,39^ This is an area requiring future research.

### Behavioural/psychosocial protective factors and brain age gap

We found statistically significant associations between behavioural/psychosocial factors and brain age, both cross-sectionally and longitudinally. At baseline, those above the group median of behavioural/psychosocial protective factors had brains that appeared about 2.7 years younger than those below the group median of behavioural/psychosocial protective factors. This effect was significant, accounting for chronic pain stage and socioenvironmental risk. Our linear mixed-effects model analysis revealed a significant and negative association between behavioural/psychosocial protective factors and brain age (*β* = −0.71, *P*  *=* 0.003), suggesting that the higher number of behavioural/psychosocial protective factors is predictive of lower brain age over a two-year span. This association persisted even after adjusting for potential confounders, including chronological age, sex, study site, comorbidities, image quality, chronic pain stage and socioenvironmental risk.

We also included in our model the random intercepts for participants, which allowed us to account for the inherent individual variability in baseline brain age. There was insufficient evidence of an interaction between time and behavioural/psychosocial protective factors (*P*  *=* 0.062), but the pattern suggests having more behavioural/psychosocial protective factors possibly slows brain ageing (about 0.25 years for each 1 unit increase in the behavioural/psychosocial protective factor). Thus, our results suggest that individuals who have more behavioural/psychosocial protective factors have brains that appear younger during middle to older age and experience slower brain ageing over time. If this pattern holds over longer timeframes, as time progresses, the buffering effect of behavioural/psychosocial protective factors on brain ageing could become more pronounced.

Our previous research investigating behavioural/psychosocial protective factors on pain-related brain structures showed differing complex relationships across different brain areas.^7^ Previous work indicates that behavioural/psychosocial protective factors buffer pain symptoms.^7^ Our findings underscore the potential buffering role these protective factors have in mitigating brain ageing processes.

There are several factors to consider that might explain the brain-buffering effect we found. Our measure of behavioural/psychosocial protective factors combined measures of tobacco use, waist circumference, optimism, positive and negative affect, perceived stress, social support, and sleep impairment. Previous work suggests smoking is associated with higher brain age,^129^ higher adiposity is associated with gray matter atrophy,^130^ mood, sleep, and affect are associated with brain age,^93,131^ and loneliness is also associated with higher brain age.^124^

Using the UK Biobank dataset, researchers demonstrated the protective effect on a ‘last in, first out’ brain network of various behavioural/psychosocial factors, some of which overlapped with our measures (e.g. sleep, body size measurements, smoking, socialization).^132^ While some previous work used machine learning models of brain age, the studies applying brain age models were all cross-sectional. Our findings are particularly novel as the measures behavioural/psychosocial protective status are based on empirically supported factors using standardized measures and established clinical norms and ranges. Importantly, each of the factors is modifiable. Our results, and the work of others, suggest a clinical focus on modifiable behavioural/psychosocial protective factors might help protect the brain against the effects of ageing.

Mechanisms by which behavioural/psychosocial protective factors might protect against brain ageing are varied. It is possible many of the factors included in our measure affect the brain through stress-adaptive systems. Behavioural/psychosocial protective factors have been associated with reduced risk of mortality in individuals with widespread pain^31^ and higher levels of individual health status, indicating lower levels of allostatic load in individuals with chronic musculoskeletal pain as demonstrated by a lower multisystem composite,^65,133^ longer telomeres,^61^ and differing pain-related brain structure.^7^ Thus, individuals who have more behavioural/psychosocial protective factors might have stress system functioning protection against neurobiological ageing and age-related brain pathology.^134^

### Strengths, limitations, and future directions

Our study has several strengths. First, we applied an externally validated machine learning model of brain age, which allows a holistic metric of brain structure, to both cross-sectional and longitudinal data. Longitudinal brain age studies are limited.^30^ Our data show that predicted brain age increased on average over a two-year span. This is an important validation of brain age as a metric that can capture brain ageing over time, providing further validation of the utility of machine learning models of brain age for longitudinal analyses. Other strengths of the study include a sample size of 197 participants with diverse representation of community-dwelling non-Hispanic Black and non-Hispanic White adults from two different regions in the United States—Birmingham, Alabama and Gainesville, Florida. Additionally, we adjusted analyses for image quality. The analyses incorporated a ‘whole person’ approach by including relevant sociodemographic measures, consideration of socioenvironmental risk factors, and inclusion of clinically relevant and modifiable behavioural/psychosocial factors.

Limitations also warrant acknowledgement. First, three of the four questions in the chronic pain stage measure were specific to the knee; thus, frequency, intensity, and duration of pain at other body sites were not captured. Second, it is possible that some participants have undiagnosed or unreported medical conditions that affect brain age that were not identified in the health history assessment. Third, although we included study site and image quality as covariates in analyses, the MRI scanners, though similar make and model, differed in the number of coils/channels, with UAB having 8 channels and UF having 32 channels. Fourth, we also acknowledge that there might be other socioenvironmental and behaviouural/psychosocial factors and associated measures warranting inclusion. We were limited to what was available in the UPLOAD-2 study dataset. Additionally, positive and negative trait effects were included in the behavioural/psychosocial protective factor. Our more recent work, consistent with the diathesis stress model, demonstrates that positive and negative trait effects are a predisposing biologically based factor. Thus, a different modelling approach could be even more informative.^135^ A fifth limitation, as this was an analysis of existing data, is that we did not have all the factors influencing brain age and ageing. In fact, capturing all the factors that influence global brain structure would be quite difficult.

There are several other opportunities to address in future research. Longitudinal investigations of brain ageing across multiple time points will enhance our understanding of change over time and the identification of factors influencing those changes. There is also a need to model the linear and nonlinear brain age gap, and not just predicted brain age changes over multiple time points. Other empirically supported socioenvironmental and behavioural/psychosocial factors warrant consideration, and the identification of the most predictive factors might further improve modelling. While independent research is demonstrating the utility of machine-learning MRI metrics such as predicted brain age for clinical questions and populations, there are multiple methods using various modalities of neuroimaging (e.g. functional MRI^136^ and EEG^137^). Work should continue to validate and compare the external validity and clinical utility of machine-learning imaging methods. Lastly, risk and protective factors can contribute towards resilience across all levels of functioning (biological to environmental). With researchers’ efforts aligning phrasing and modeling consistently, comparisons and integrations across findings will help advance the science of resilience.^138^

## Conclusions

Applying standardized measures and clinical norms and ranges, we evaluated the biological interface of chronic pain severity, socioenvironmental risk, and behavioural/psychosocial factors.

As socioenvironmental and behavioural/psychosocial factors can reduce or increase risk of poor health outcomes, we show a combined ‘whole person’ approach associates with brain age differences in individuals with chronic musculoskeletal pain. Modifiable behavioural/psychosocial protective factors show the same buffering benefits neurobiologically that we have reported in a clinical multisystem composite.^79^

Our results indicate that while chronic pain is correlated with overall brain structure, socioenvironmental and behavioural/psychosocial factors appear to play a more significant role. Given that the behavioural/psychosocial factors correlate with brain age over time and are potentially modifiable, the protective factors provide a set of potential clinical targets (e.g. sleep, smoking, social support) for interventions that might reduce brain ageing in middle and old age within and without the context of chronic pain.

## Supplementary Material

fcaf344_Supplementary_Data

## Data Availability

The data supporting the findings of this study can be obtained from the corresponding author upon reasonable request. DeepBrainNet Python code used is available here: https://github.com/tannerjared/DeepBrainNet. The R code used for statistical analyses is available here: https://github.com/tannerjared/ProtectiveBAG.
