# Supplementary material for: More than chronic pain: behavioural and psychosocial protective factors predict lower brain age in adults with/at risk of knee osteoarthritis over two years
Source: Brain Commun. 2025 Sep 11;7(5):fcaf344. doi: 10.1093/braincomms/fcaf344 (PMC12465110; doi:10.1093/braincomms/fcaf344)
Supplement: fcaf344_Supplementary_Data [file fcaf344_supplementary_data.docx]

**Supplemental Data, Tables, and Figures**

**Data**

**Supplemental Results**

**Brain age change (time point 1 < time point 2 vs. time point 2 < time point 1) and baseline characteristics.**

Total *n* = 128

Number and percent of participants where brain age time point 1 < brain age time point 2 (increase): 88/128 (68.75%)

Percent of participants where brain age time point 1 > brain age time point 2 (decline): 40/128 (31.25%)

**T tests for group (decline versus increase) differences in baseline measures**

Age: *t* = 0.61, *P* = 0.544

Comorbidities: *t* = 0.27, *P* = 0.790

Image Quality Rating: *t* = 0.17, *P* = 0.866

Chronic pain stage: *t* = 0.745, *P* = 0.457

SE risk: *t* = 0.35, *P* = 0.726

Behavioral/psychosocial protective factors: *t* = -1.70, *P* = 0.093

**Chi-squared tests for group (decline versus increase) differences in baseline measures**

Sex: *χ^2^* = 0.02, *P* = 0.898

Site: *χ^2^* = 1.14, *P* = 0.286

**Tables**

**Supplementary Table 1. Regression Predicting Brain Age Gap from Chronic Pain Stage**

| **Predictor** | ***b*** | **95% CI** | ***beta*** | **95% CI** | **Unique *R^2^*** | **95% CI** | ***r*** | **Fit** |
| --- | --- | --- | --- | --- | --- | --- | --- | --- |
| (Intercept) | 27.32* | [3.37, 51.27] |  |  |  |  |  |  |
| IQR | -0.30* | [-0.59, -0.02] | -0.17 | [-0.33, -0.01] | 0.02* | [-0.02, 0.06] | -0.19** |  |
| Sex | -3.54** | [-5.49, -1.60] | -0.25 | [-0.39, -0.11] | 0.06** | [-0.00, 0.12] | -0.28** |  |
| Site | -1.25 | [-3.38, 0.89] | -0.09 | [-0.24, 0.06] | 0.01 | [-0.01, 0.03] | -0.01 |  |
| Comorbidities | -0.00 | [-0.87, 0.86] | -0.00 | [-0.15, 0.14] | 0.00 | [-0.00, 0.00] | 0.05 |  |
| CPS | 0.67* | [0.04, 1.30] | 0.15 | [0.01, 0.29] | 0.02* | [-0.02, 0.06] | 0.17* |  |
|  |  |  |  |  |  |  |  | *R^2^*  = 0.129** |
|  |  |  |  |  |  |  |  | 95% CI [0.04,0.20] |

*Note.* *N* = 197. *b* = unstandardized regression weight. *beta* = standardized regression weight. Unique *R^2^* = semipartial correlation squared. *r* = zero-order correlation. CI = confidence interval. IQR = Image Quality Rating; CPS = Chronic Pain Stage

* indicates *P <* 0.05. ** indicates *P <* 0.01.

**Supplementary Table 2. Regression Predicting Brain Age Gap from Socioenvironmental Risk**

| **Predictor** | ***b*** | **95% CI** | ***beta*** | **95% CI** | **Unique *R^2^*** | **95% CI** | ***r*** | **Fit** |
| --- | --- | --- | --- | --- | --- | --- | --- | --- |
| (Intercept) | 21.38 | [-2.25, 45.02] |  |  |  |  |  |  |
| IQR | -0.24 | [-0.52, 0.04] | -0.13 | [-0.29, 0.02] | 0.01 | [-0.02, 0.04] | -0.19** |  |
| Sex | -3.29** | [-5.19, -1.38] | -0.23 | [-0.37, -0.10] | 0.05** | [-0.01, 0.11] | -0.28** |  |
| Site | -0.92 | [-3.00, 1.17] | -0.07 | [-0.22, 0.08] | 0.00 | [-0.01, 0.02] | -0.01 |  |
| Comorbidities | 0.13 | [-0.69, 0.94] | 0.02 | [-0.12, 0.16] | 0.00 | [-0.00, 0.01] | 0.05 |  |
| SE Risk | 1.00** | [0.48, 1.53] | 0.26 | [0.12, 0.39] | 0.06** | [0.00, 0.12] | 0.31** |  |
|  |  |  |  |  |  |  |  | *R^2^*  = 0.172** |
|  |  |  |  |  |  |  |  | 95% CI [0.07,0.25] |

*Note.* *N* = 197. *b* = unstandardized regression weight. *beta* = standardized regression weight. Unique *R^2^* = semipartial correlation squared. *r* = zero-order correlation. CI = confidence interval. IQR = Image Quality Rating; SE Risk = Socioenvironmental Risk
* indicates *P <* 0.05. ** indicates *P <* 0.01.

**Supplementary Table 3.** **Regression Predicting Brain Age Gap from Behavioral/Psychosocial Protective Factors**

| **Predictor** | ***b*** | **95% CI** | ***beta*** | **95% CI** | **Unique *R^2^*** | **95% CI** | ***r*** | **Fit** |
| --- | --- | --- | --- | --- | --- | --- | --- | --- |
| (Intercept) | 30.72** | [7.85, 53.59] |  |  |  |  |  |  |
| IQR | -0.23 | [-0.51, 0.05] | -0.13 | [-0.28, 0.03] | 0.01 | [-0.02, 0.04] | -0.19** |  |
| Sex | -3.41** | [-5.30, -1.53] | -0.24 | [-0.37, -0.11] | 0.05** | [-0.00, 0.11] | -0.28** |  |
| Site | -1.23 | [-3.30, 0.83] | -0.09 | [-0.24, 0.06] | 0.01 | [-0.01, 0.03] | -0.01 |  |
| Comorbidities | -0.13 | [-0.95, 0.70] | -0.02 | [-0.16, 0.12] | 0.00 | [-0.00, 0.01] | 0.05 |  |
| Behavioral/Psychosocial Protective Factors | -0.69** | [-1.01, -0.37] | -0.29 | [-0.43, -0.16] | 0.08** | [0.01, 0.14] | -0.32** |  |
|  |  |  |  |  |  |  |  | *R^2^*  = 0.185** |
|  |  |  |  |  |  |  |  | 95% CI [0.08,0.26] |

*Note.* *N* = 197. *b* = unstandardized regression weight. *beta* = standardized regression weight. Unique *R^2^* = semipartial correlation squared. *r* = zero-order correlation. CI = confidence interval. IQR = Image Quality Rating
* indicates *P <* 0.05. ** indicates *P <* 0.01.

**Supplementary Table 4. Linear Mixed Model Predicting Brain Age Over Two Years from Chronic Pain Stage**

| **Parameter** | ***Coefficient*** | **SE** | **95% CI** | **t** | **p** |
| --- | --- | --- | --- | --- | --- |
| **Fixed Effects** | | | | | |
| (Intercept) | 34.21 | 10.88 | [12.87, 55.54] | 3.14 | 0.002 |
| Time** | 1.44 | 0.36 | [0.72, 2.15] | 3.94 | 0.000 |
| Sex** | -3.64 | 1.28 | [-6.14, -1.13] | -2.85 | 0.005 |
| Age** | 0.86 | 0.07 | [0.72, 1.00] | 12.31 | 0.000 |
| Site | -0.82 | 1.26 | [-3.30, 1.66] | -0.65 | 0.517 |
| Comorbidities | 0.36 | 0.60 | [-0.83, 1.54] | 0.59 | 0.557 |
| IQR** | -0.30 | 0.11 | [-0.53, -0.08] | -2.69 | 0.008 |
| CPS* | 0.84 | 0.40 | [0.06, 1.63] | 2.11 | 0.037 |
| **Random Effects** | | | | | |
| Participant (Intercept) | 0.60 | 0.04 | [0.52, 0.69] | -- | -- |
| Residual (Observations) | 0.28 | 0.02 | [0.25, 0.32] | -- | -- |

*Note.* *N* = 128. SE = standard error of the model estimates. IQR = Image Quality Rating; CPS = Chronic Pain Stage; Participant (Intercept) = the standard deviation of the random intercepts across participants; Residual (Observations) = Residual standard deviation.
* indicates *P <* 0.05. ** indicates *P <* 0.01.

**Supplementary Table 5. Linear Mixed Model Predicting Brain Age Over Two Years from Socioenvironmental Risk**

| **Parameter** | ***Coefficient*** | **SE** | **95% CI** | **t** | **p** |
| --- | --- | --- | --- | --- | --- |
| **Fixed Effects** | | | | | |
| (Intercept) | 31.55 | 11.05 | [9.89, 53.21] | 2.86 | 0.005 |
| Time** | 1.44 | 0.36 | [0.72, 2.15] | 3.94 | 0.000 |
| Sex* | -3.13 | 1.29 | [-5.67, -0.59] | -2.42 | 0.017 |
| Age** | 0.89 | 0.07 | [0.75, 1.04] | 12.45 | 0.000 |
| Site | -0.53 | 1.26 | [-3.01, 1.94] | -0.42 | 0.673 |
| Comorbidities | 0.61 | 0.58 | [-0.54, 1.75] | 1.04 | 0.302 |
| IQR** | -0.30 | 0.11 | [-0.53, -0.08] | -2.70 | 0.008 |
| SE Risk* | 0.84 | 0.36 | [0.13, 1.56] | 2.32 | 0.022 |
| **Random Effects** | | | | | |
| Participant (Intercept) | 0.60 | 0.04 | [0.52, 0.69] | -- | -- |
| Residual (Observations) | 0.28 | 0.02 | [0.25, 0.32] | -- | -- |

*Note.* *N* = 128. SE = standard error of the model estimates. IQR = Image Quality Rating; SE Risk = Socioenvironmental Risk; Participant (Intercept) = the standard deviation of the random intercepts across participants; Residual (Observations) = Residual standard deviation.
* indicates *P <* 0.05. ** indicates *P <* 0.01.

**Supplementary Table 6. Linear Mixed Model Predicting Brain Age Over Two Years from Behavioral/Psychosocial Protective Factors**

| **Parameter** | ***Coefficient*** | **SE** | **95% CI** | **t** | **p** |
| --- | --- | --- | --- | --- | --- |
| **Fixed Effects** | | | | | |
| (Intercept) | 41.74 | 10.60 | [20.96, 62.51] | 3.94 | 0.000 |
| Time* | 1.43 | 0.36 | [0.72, 2.15] | 3.94 | 0.000 |
| Sex** | -3.22 | 1.21 | [-5.59, -0.85] | -2.67 | 0.009 |
| Age** | 0.93 | 0.07 | [0.79, 1.06] | 13.68 | 0.000 |
| Site | -0.86 | 1.19 | [-3.20, 1.47] | -0.73 | 0.470 |
| Comorbidities | 0.30 | 0.56 | [-0.79, 1.40] | 0.55 | 0.586 |
| IQR** | -0.30 | 0.11 | [-0.52, -0.08] | -2.64 | 0.009 |
| Behavioral/Psychosocial Protective Factors** | -0.96 | 0.21 | [-1.38, -0.55] | -4.54 | 0.000 |
| **Random Effects** | | | | | |
| Participant (Intercept) | 0.56 | 0.04 | [0.49, 0.65] | -- | -- |
| Residual (Observations) | 0.28 | 0.02 | [0.25, 0.32] | -- | -- |

*Note.* *N* = 128. SE = standard error of the model estimates. IQR = Image Quality Rating; Participant (Intercept) = the standard deviation of the random intercepts across participants; Residual (Observations) = Residual standard deviation.
* indicates *P <* 0.05. ** indicates *P <* 0.01.

**Figures**

**Supplementary Figure 1. Violin plot showing brain age gap with socioenvironmental risk groups (Aim 1b)**

**
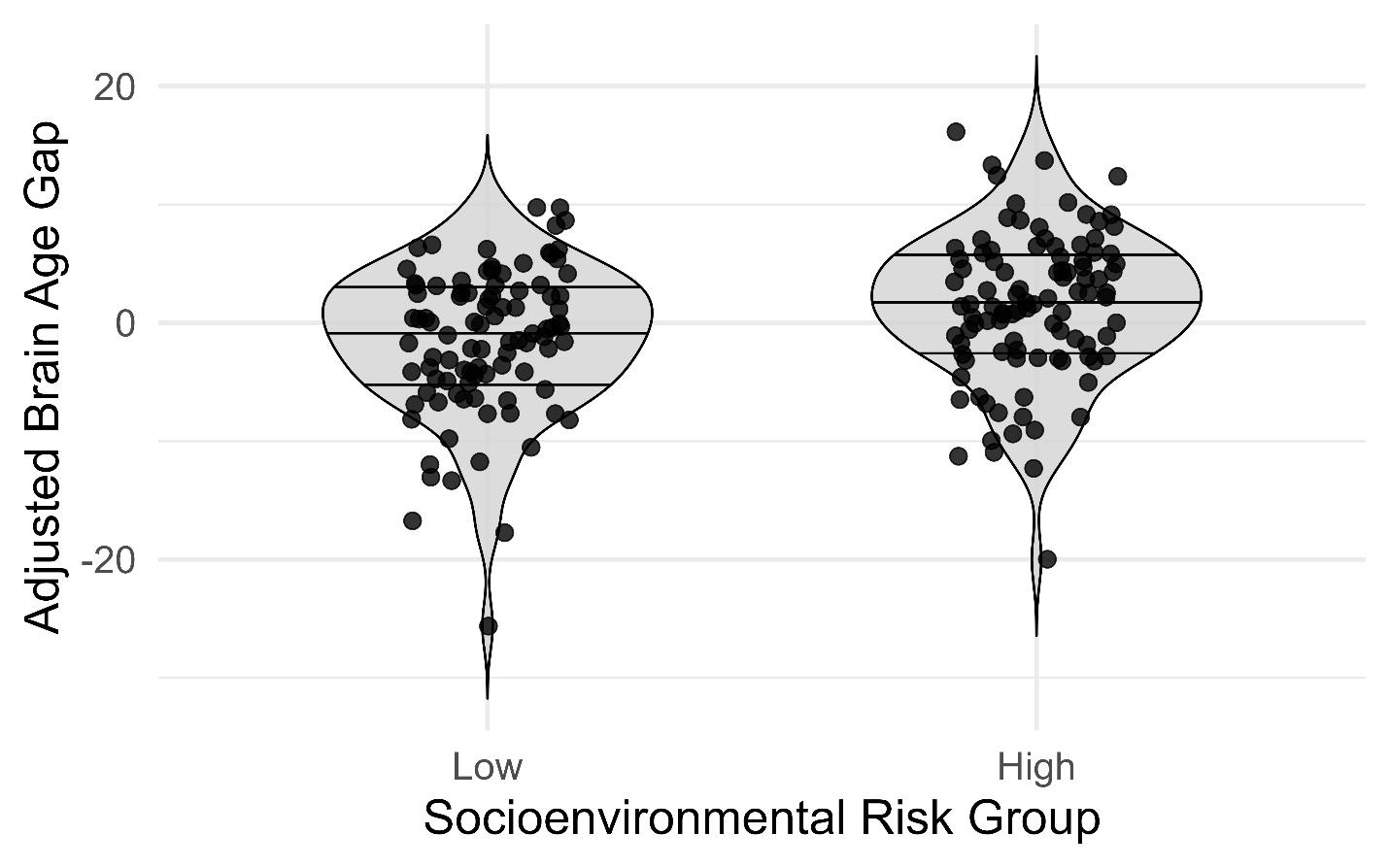
**

Adjusted Brain Age Gap is corrected for sex, study site, comorbidities, and image quality rating. The socioenvironmental risk (SE risk) groups are based on a median split. The group with higher SE risk had an adjusted brain age gap (BAG) 2.99 years ‘older’ than the group with low risk factors (Model ANOVA *F(5,191)* = 7.068, *P <* 0.001; group t = 3.273, p = 0.001, *η_p_^2^* = 0.05 [0.01, 0.11], n = 197). Individual circles are the adjusted BAG values for each participant.

**Supplementary Figure 2.** **Violin plot showing brain age gap with behavioral/psychosocial protective factors groups (Aim 1c)**


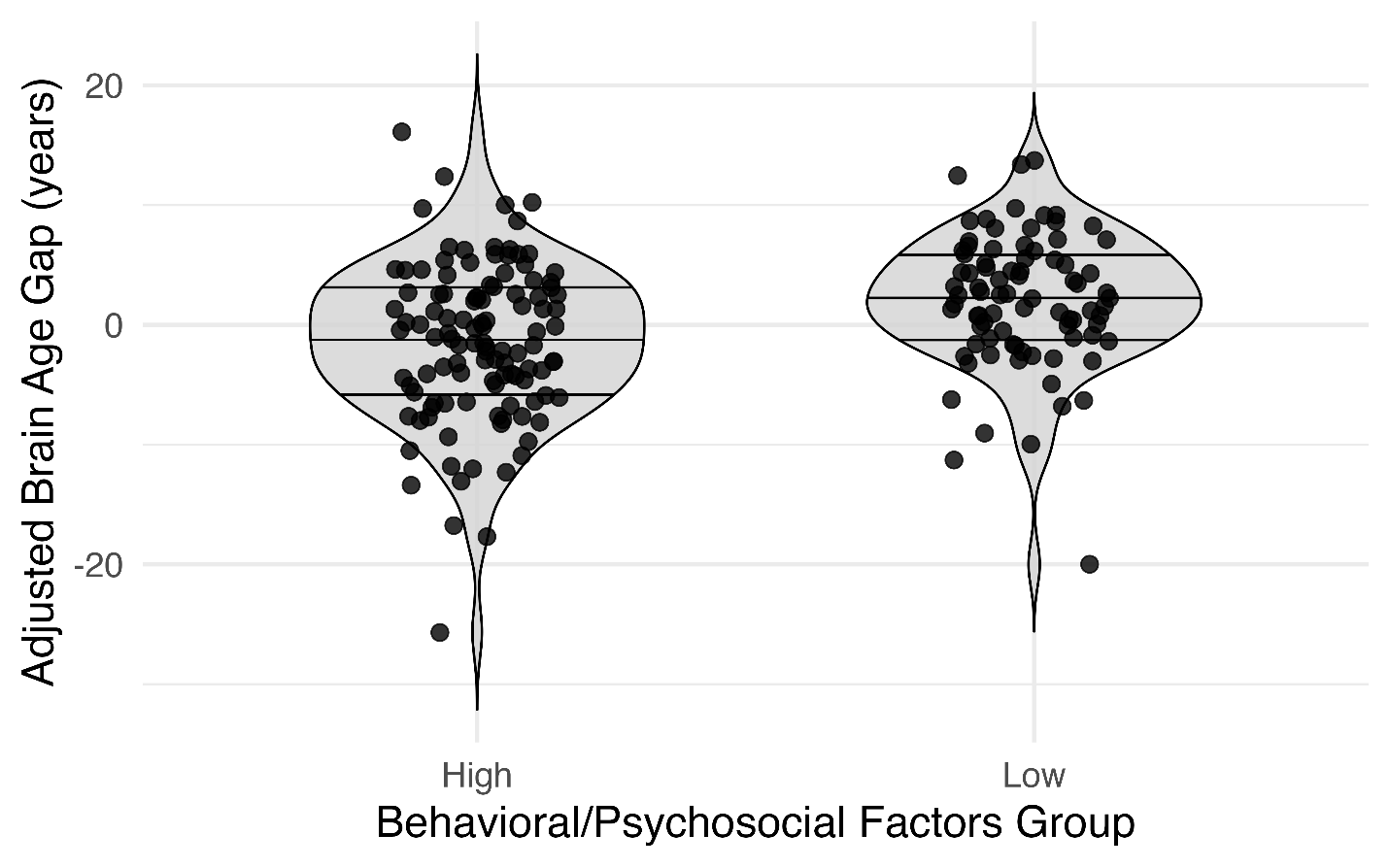


Adjusted Brain Age Gap is corrected for sex, study site, comorbidities, and image quality rating. Behavioral/Psychosocial protective factors groups were based on the median split. The group with higher behavioral/psychosocial protective factors had an adjusted brain age gap (BAG) 3.78 years ‘younger’ than the group with lower protective factors (Model ANOVA *F(5,191)* = 8.426, *P <* 0.001; group t = -4.10, p < 0.001; *η_p_^2^* = 0.08 [0.03, 0.15], n = 197). Individual circles are the adjusted BAG values for each participant.

**Supplementary Figure 3. Spaghetti plot showing brain ages over 2 years with three behavioral/psychosocial protective factors groups (Aim 2)**


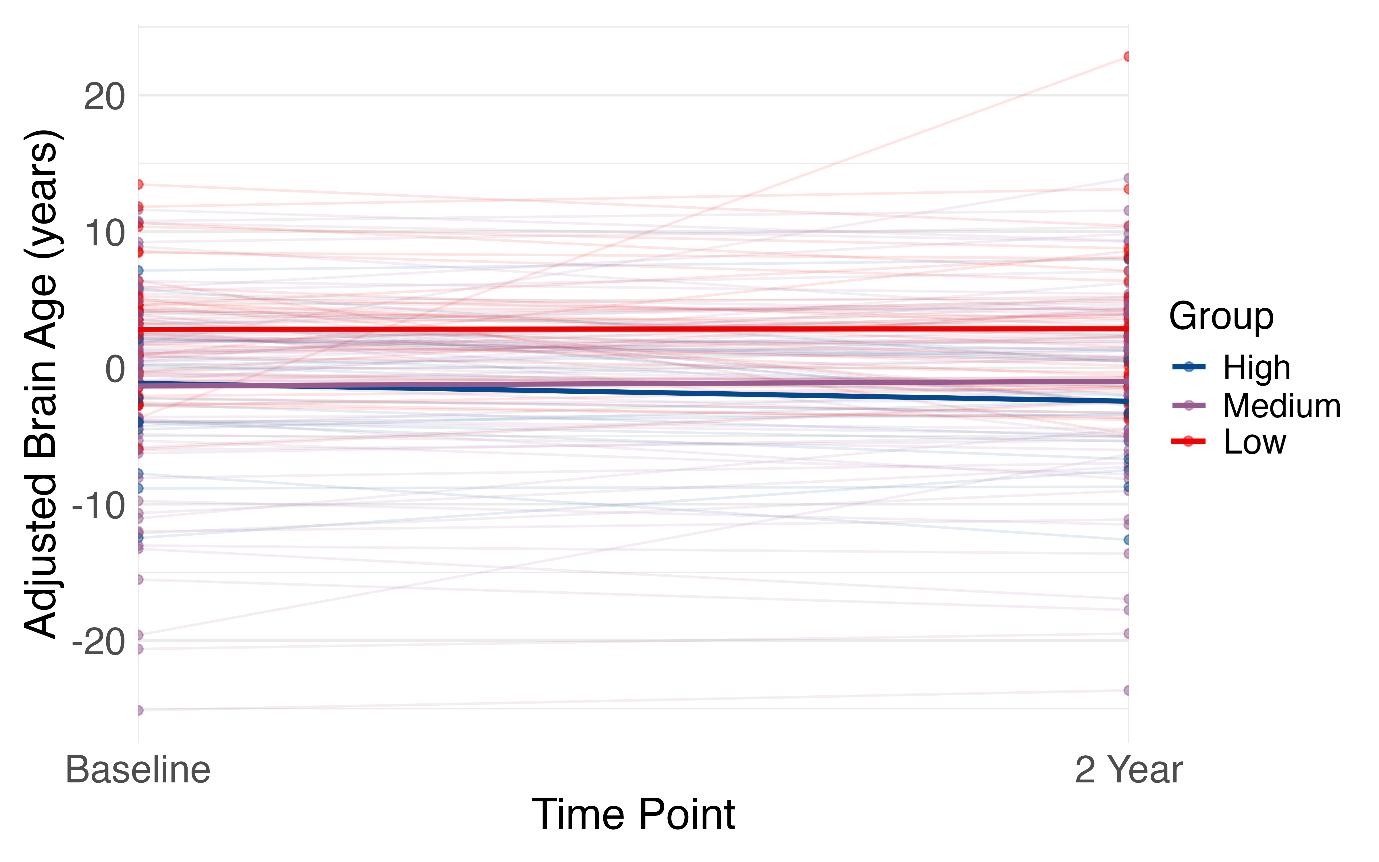


This figure depicts the changes in adjusted brain ages over the two years of the study (n = 128). Brain age is adjusted for chronological age, sex, study site, comorbidities, image quality rating at both time points, chronic pain stage, and socioenvironmental risk.

The groups are based on the behavioral/psychosocial protective factors groups. They were created *post hoc* to visualize the behavioral/psychosocial protective factors and behavioral/psychosocial protective factors * time effects. Behavioral/Psychosocial protective factors groups: Low = ≤25^th^ percentile, Medium = >25^th^ and <75^th^ percentile, High = ≥75^th^ percentile.

This figure shows individuals with the lowest number of behavioral/psychosocial protective factors (≤25^th^ percentile) have older brain ages at both time points than the other two groups. Additionally, the pattern of the highest behavioral/psychosocial protective factors group suggests that these individuals on average experience no brain aging and even reduction in predicted brain ages over the two years. That there might be increasing differences between low and high behavioral/psychosocial protective factors groups over time is suggested by the reported findings in the Aim 2 *linear mixed model*: time * protective factors effect (*P* = 0.062).
